# Predictors of differences in health services utilization for children in Nigerian communities

**DOI:** 10.1016/j.ypmed.2016.12.035

**Published:** 2017-03

**Authors:** Victor T. Adekanmbi, Sulaimon T. Adedokun, Sian Taylor-Phillips, Olalekan A. Uthman, Aileen Clarke

**Affiliations:** a NIHR Collaboration for Leadership in Applied Health Research and Care, West Midlands (CLAHRC WM), University of Warwick Medical School, Coventry, United Kingdom; b Department of Demography and Social Statistics, Obafemi Awolowo University, Ile-Ife, Nigeria; c Warwick-Centre for Applied Health Research and Delivery (WCAHRD), Division of Health Sciences, University of Warwick Medical School, Coventry, United Kingdom

**Keywords:** Health services utilization, Under-fives, Variations, Contextual factors, Nigeria

## Abstract

Health service utilization is an important component of child health promotion. Evidence shows that two-thirds of child deaths in low and middle income countries could be prevented if current interventions were adequately utilized. Aim of this study was to identify determinants of variation in health services utilization for children in communities in Nigeria. Multivariable negative binomial regression model attempting to explain observed variability in health services usage in Nigerian communities was applied to the 2013 Nigeria Demographic and Health Survey data. We included the index of maternal deprivation, gender of child, community environmental factor index, and maternal health seeking behaviour, multiple childhood deprivation index and ethnicity diversity index as the independent variables. The outcome variable was under-fives' hospital attendance rates for acute illness. Of the 7577 children from 896 communities in Nigeria that were sick 1936 (25.6%) were taken to the health care facilities for treatment. The final model revealed that both multiple childhood deprivation (incidence rate ratio [IRR] = 1.23, 95% confidence interval [CI] 1.12 to 1.35) and children living in communities with a high ethnic diversity were associated with higher rate of health service use. Maternal health seeking behaviour was associated with a significantly lower rate of health care service use. There are significant variations in health services utilization for sick children across Nigeria communities which appear to be more strongly determined by childhood deprivation factors and maternal health seeking behaviour than by health system functions.

## Highlights

• Predictive model of factors associated with health services use in Nigeria
• Childhood deprivation was associated with a higher rate of health care services use.
• Maternal health seeking behaviour was associated with lower health service use.

## 1 Background

Health service utilization is an important component of child health promotion. Evidence shows that two-thirds of child deaths in low and middle income countries (LMICs) could be prevented if current interventions were adequately utilized (Jones et al., 2003). Utilization of such interventions in high childhood mortality regions remains low (Winter et al., 2015). Nigeria has high childhood mortality, in 2015, under-five mortality rate (UFMR) was 109 per 1000 live births with only a 2.7% annual rate of reduction (ARR) expected over the period of 25 years from the year 1990 (UNICEF, 2015). In an attempt to address this problem, the Federal Ministry of Health (FMOH) in Nigeria developed a child health policy which introduced interventions covering areas such as perinatal and neonatal health, young child feeding, major childhood illness, HIV/AIDS, school health and injury protection (Federal Ministry of Health, 2006). One of the objectives of these interventions was to increase health service utilization through the removal of obstacles to the uptake of health services for children (Federal Ministry of Health, 2006). Reports, however, indicate that public health service facilities have witnessed decreased utilization from mid-1980s to year 2010 (Federal Ministry of Health, 2010).

Currently, there is increasing interest in the levels of performance of the interventions adopted in the Nigeria National Strategic Health Development Plan (NSHDP) especially in the area of childhood health outcomes and health services utilization (Federal Ministry of Health, 2010). Although most communities in Nigeria have low health services usage, it is unclear whether lower service utilization arises from low maternal health seeking behaviour, low parental levels of education, weak regional health system or from other demographics such as ethnicity, sex or deprivation.

Recent efforts at improving health services utilization are shifting to the community. The role of the community in promoting child health has been recognized by the international community and led to the establishment of the Integrated Management of Childhood Illness (IMCI) initiative which has three key components. One of these is improvement in family and community care practices (Hill et al., 2004). In line with this, the FMOH of Nigeria highlighted the involvement of family and community as part of its strategic plan for health service improvement (Federal Ministry of Health, 2010). However, the successful engagement of the community hinges on a better understanding of the differing factors which operate in various communities. Such factors may contribute to the differentials in health service utilization but there have been relatively few studies to date which have examined these factors (Sule et al., 2013, Nzioki et al., 2015, Emmanuel et al., 2013, Wolff, 1993, Kenny et al., 2015, Benova et al., 2015, Geldsetzer et al., 2014, Uzochukwu et al., 2008, Merrin et al., 2010). The aim of this study was to examine factors which predict differentials in health service utilization for children in Nigerian communities.

## 2 Methods

### 2.1 Study design

This study was based on secondary analyses of cross-sectional population-based data from the 2013 Nigeria Demographic and Health Survey (DHS).

### 2.2 Setting

Nigeria covers a total area of about 923,768 km^2^. It is the thirty-second largest country in terms of land mass and the most populous country in Africa with a recent estimate of its population as 140,431,790 (NPC, ICF International, 2013). About 67.8% of the population live in rural areas and 32% in urban areas. There are 374 identifiable ethnic groups in Nigeria with varying languages, customs and cultures (NPC, ICF International, 2013). The largest ethnic groups are the Yoruba, Hausa/Fulani and Igbo which account for about 68% of the population (NPC, ICF International, 2013). Available statistics indicate that about 8% of the population are categorised as poor, 34% as lower class, 25% as lower middle class, 18% as upper middle class, 8% as lower upper class and 3% as upper class (Nigeria Population Distribution by Socioeconomic Class, 2015).

### 2.3 Sampling technique

The 2013 DHS (NPC, ICF International, 2013) was conducted in Nigeria to collect data on demographic, environmental, socioeconomic, and health issues (family planning, infertility, nutritional and health status of children, their mothers and the fathers) from a nationally representative sample of 39,902 women aged 15–49 years and 18,229 men aged 15–59 years in 38,904 households (NPC, ICF International, 2013). The survey used a three-stage cluster sampling technique. The country was stratified into 36 States and the Federal Capital Territory (FCT), Abuja making 37 districts in total. The primary sample unit (PSU) was based on 2006 General Population and Housing Census enumeration areas (EAs). The first stage involved selecting 896 localities (clusters). In the second stage, one EA was randomly selected from most localities. A total of 904 EAs were selected, with 372 in urban areas and 532 in rural areas (NPC, ICF International, 2013). The third stage involved random selection of a fixed number of 45 households in every urban and rural geographical area.

### 2.4 Data collection

Data collection procedures have been published elsewhere (NPC, ICF International, 2013). Data (on demographic characteristics, wealth, anthropometry, female genital cutting and awareness of HIV/AIDS, knowledge of HIV prevention, sexual behaviour, and domestic violence) were collected by conducting face-to-face interviews with women and men who met the eligibility criteria. Among all eligible individuals and households, participation rates were 98% for household, 98% for women and 95% for men (NPC, ICF International, 2013). Each woman was asked to provide a detailed history of all her live births in chronological order, including whether a birth was single or multiple, assigned sex of the child, date of birth, survival status, age of the child on the date of interview if alive and age at death of each live birth, if the child was not still alive.

### 2.5 Outcome variable

Hospital attendance rates for acute illness at a community level was the response variable. We focused on data for children under-five who had had an episode of diarrhea and/or fever/cough in the preceding 2 weeks before the survey and whose parents/carers sought consultation from a health care provider (either public or private).

### 2.6 Independent variables

We included the following independent variables; gender of child, community environmental factor index, maternal health seeking behaviour, multiple childhood deprivation index and ethnicity diversity index. We used composite indices because they are easier to interpret than a battery of separate indicators and because they help to construct narratives for lay and literate audiences. In addition, they reduce the visible size of a set of indicators without dropping underlying information. Furthermore, multidimensional concepts like welfare, well-being, human development, environmental sustainability, industrial competitiveness and so on cannot be adequately represented by individual indicators (OECD, 2008).

#### 2.6.1 Childhood deprivation index

We used a childhood deprivation index previously described in a study by Uthman (2009). The childhood deprivation index in this study was operationalized with a principal component comprised of the proportion of children with low birth weight, not breast fed, with short birth interval (< 24 months), high number of under-fives in the household and children with high birth order. A standardized score with mean 0 and standard deviation of 1 was generated from this index; with higher scores indicative of higher childhood deprivation (Uthman, 2009).

#### 2.6.2 Maternal deprivation index

Maternal deprivation comprised of the proportion of mothers who are non-literate, unemployed, residing in rural areas and living below the poverty level (asset index < 20% poorest quintile).

#### 2.6.3 Community environmental factor index

This was derived using principal component analysis on 3 variables that included proportion of children in households with access to safe water, proper sanitation, and low pollution cooking fuel. A standardized score with mean 0 and standard deviation of 1 was generated with higher scores indicative of better and cleaner environmental status.

#### 2.6.4 Maternal health seeking behaviour index (MHSBI)

This was operationalized with a principal component analysis comprised of the proportion of respondents: with a health card, who attended ante natal care clinic and received tetanus vaccine during pregnancy, with the child's delivery in the hospital and with child received at least one dose of vaccination. A standardized score with mean 0 and standard deviation of 1 was generated from this index; with higher scores indicative of better MHSBI.

#### 2.6.5 Ethnicity diversity index

The ethnicity of the children was computed by using an ethnicity diversity index. This index was created using a formula (Eq. (1) below) which captures both the number of different ethnic groups in an area and the relative representation of each group (Vyas and Kumaranayake, 2006).

(1) $$\mathrm{Ethnic diversity index}=1-\Sigma_{i=1}^{n}{\left[\frac{\mathrm{xi}}{y}\right]}^{2}$$

where: *x*_*i*_ = population of ethnic group i of the area, y = total population of the area, n = number of ethnic groups in the area.

Scores can range from 0 to approximately 1. For clarity of interpretation, each diversity index is multiplied by 100; the higher the index score, the greater the diversity in the area. If an area's entire population belongs to one ethnic group, then the area has zero diversity. An area's diversity index increases to 100 when the population is evenly divided into ethnic groups.

### 2.7 Ethical considerations

This study was based on secondary analysis of existing survey datasets from the archive of the DHS who granted us permission for use of anonymised data. The instruments and conduct of the 2013 Nigeria DHS was approved by the Institutional Review Board (IRB) of ICF Macro International in the United States and Nigeria Health Research Ethics Committee (NHREC) of the FMOH. This research is limited to the use of previously collected anonymised data.

### 2.8 Statistical analysis

To determine the number of component included in the factor analyses, we used the criterion: eigenvalues ≥ 1 and we also inspected the scree plot. Factor loadings ≥ 0.4 were judged to be significant. The results of the PCA tests are included in the online Supplementary material (tables 4, 5 and 6). A negative binomial multivariable regression model due to over-dispersion of the outcome variable was developed to explain the observed variability in health services utilization described by Harrell et al. (1996) and Freemantle et al. (2009).

Associations between health service utilization and all included independent variables were first examined at the univariable level. Gender and characteristics statistically associated with a *P* value of 0.1 at univariable level were subsequently fitted in the multivariable negative binomial regression model. Gender was included in the multivariable negative binomial regression model because we wanted to assess interaction effects of gender and other independent variables. We also fitted another multivariable negative binomial regression using backward stepwise model selection with a *P* value of 0.10 with the result similar to the previously described method (see Supplementary material table 3). *P* value of < 0.05 was used to define statistical significance. The final model was used to predict the hospital attendance rates for each community (unit of analysis derived by collapsing child level data at community level) and compared the predicted and the observed hospital attendance rates.

### 2.9 Model fit and specifications

Regression diagnostics were used to judge the goodness-of-fit of the model. They included the Likelihood Ratio test (LHR test), and the Hosmer-Lemeshow model fit test. The tolerance test, variance inflation factors (VIF), reciprocal of VIF (Tu et al., 2004, Tu et al., 2005), and estimates of adjusted R squared of the regression model were used to test for multicollinearity using the routine collin in Stata. A VIF > 10 or mean VIF > 6 represent severe multicollinearity (Hocking, 1996). There was no issue of concern regarding the regression diagnostics for model fit and multicollinearity tests. In addition, model validation evaluating potential over-fitting was carried out using bootstrapping. Briefly, 100 bootstrap samples were generated from the original datasets using a resampling technique. The original model was re-fitted in the testing datasets to estimates adjusted (corrected) estimate of the predictors. The corrected estimates of the predictors remained unchanged after bootstrapping. All statistical analyses were carried out using Stata statistical software for windows version 14 (StataCorp, 2015).

## 3 Results

### 3.1 Health services utilization for sick children and community level demographics in Nigeria

**Fig. 1:**
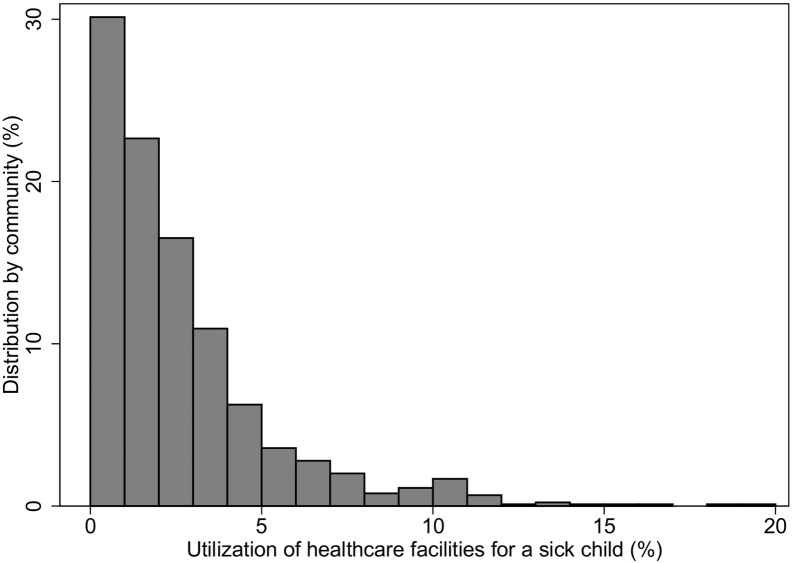
Percentage of sick children who attended health facilities by communities in Nigeria.

**Fig. 2:**
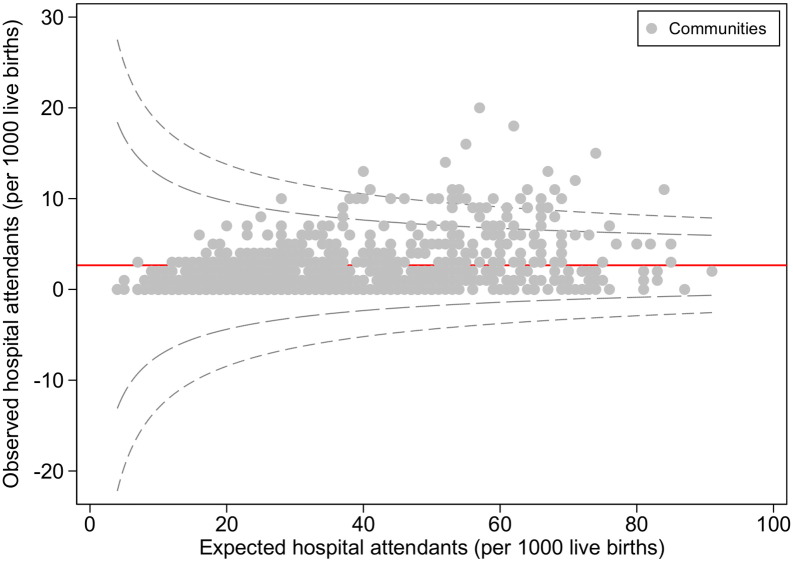
Funnel plot of health facility attendants of sick children in Nigeria, 2013. The red line represents the mean line while the inner dotted lines are 95% confidence intervals and outer dotted lines are 99.9% confidence intervals derived using the Poisson distribution.

We identified data for all 896 communities included in the 2013 Nigeria DHS. Fig. 1, Fig. 2 show the variations in rates of health service utilization across the communities in Nigeria. The average percentage of sick children taken to healthcare facilities was 2.2% (range: 0 to 20%) over the five years of the data gathering period. The mean age of the children was 26.1 months and the range was (0 to 59) months. As shown in Fig. 1, in most (807/896 = 90%) of the communities, < 5% of the children were taken to healthcare facilities when sick. There were variations in health services utilization rates for sick children between Nigerian communities using the control chart/funnel plot (see Fig. 2). The funnel plot shows that the majority of the communities had low hospital attendance' rates with a few communities having more than average attendance' rates. There were considerable variations in the parameters which made up the different indexes described above (see Table 1 for each element).

### 3.2 Community level predictors of health services utilization for sick children in Nigeria communities

#### 3.2.1 Unadjusted associations

The results of univariable (unadjusted) analyses are shown in Table 2. The childhood deprivation index, maternal deprivation index, community environmental factor index, the maternal health seeking behaviour index and ethnicity diversity index were all statistically significantly associated with health care utilization rates.

#### 3.2.2 Adjusted associations

Table 2 below shows community level predictors of health services utilization for sick children in Nigeria. The childhood deprivation index remained statistically significant after adjusting for other factors. A unit increase in standard deviation in the childhood deprivation index, was associated with a higher rate of use of health care services for more deprived children by 23% per 1000 live births (incidence rate ratio (IRR) = 1.23, 95% confidence interval [CI] 1.12 to 1.35). Likewise, a unit increase in standard deviation in the ethnicity diversity index of a community, was associated with a higher rate of health care service use for children by 0.5% per 1000 live births (IRR = 1.005, 95% CI 1.002 to 1.007). Higher maternal health seeking behaviour index was associated with a lower rate of usage of health services for children such that for every unit increase in standard deviation in the maternal health seeking behaviour index of a community, rate of use of health care services for children was lower by 36% per 1000 live births.

#### 3.2.3 Interaction effects

**Fig. 3:**
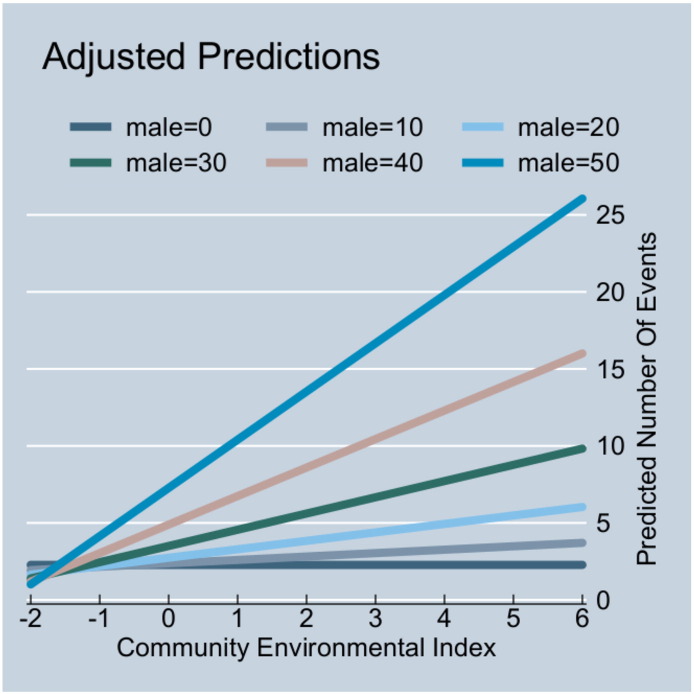
Interaction between percentage of male children in the community, community environmental factor index and health services utilization for children.

There were statistically significant interaction effects between the percentage of male children in the community, the community environmental factor index and maternal health seeking behaviour. There was a statistically significant positive interaction term for the association between the percentage of male children in the community and the community environmental factor index; suggesting that the higher the percentage of male children in the community, the greater (more positive) the effect of the community environmental factor on health service utilization. This effect is shown in Fig. 3. The community environmental factor index does not change with health service utilization for a community with only female children, but as the percentage of male children becomes higher, the community environmental factor index has a stronger positive association with health services utilization.

**Fig. 4:**
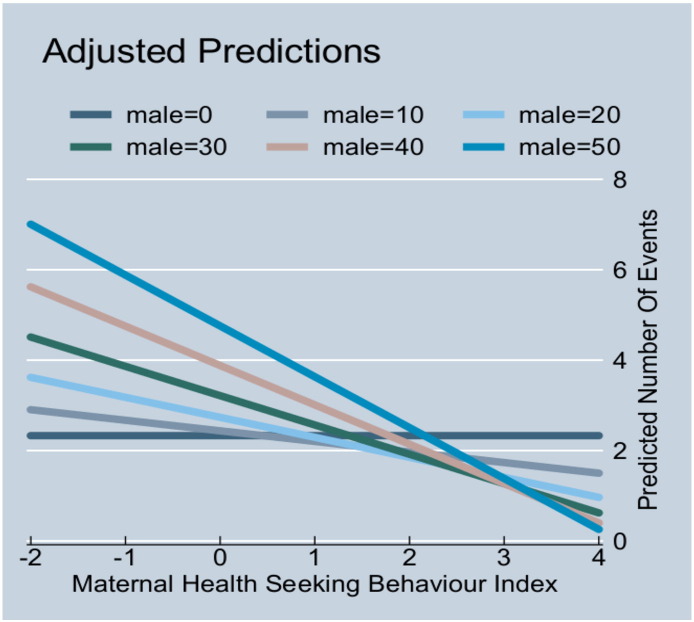
Interaction between percentage of male children in the community, maternal health seeking behaviour, and health services utilization for children.

There was also a statistically significant negative interaction term for the association between the percentage of male children in the community and maternal health seeking behaviour; suggesting that the higher the percentage of male children in the community, the more negative the association of maternal health seeking behaviour on health services utilization as shown in Fig. 4.

### 3.3 Model validation

After bootstrapping, the estimated coefficients of the predictors remained unchanged indicating that the original model was not over-fitted. In other words, there was no change in the estimated coefficients in the model after penalizing the original model.

## 4 Discussion

This study examined the factors associated with health services utilization for sick children at the community level in Nigeria. Our study shows that children with high multiple childhood deprivation indices have a higher likelihood of health services usage compared to those with lower childhood deprivation index scores. These findings are consistent with findings of previous studies (Kassile et al., 2014, Kante et al., 2015, Van den Broeck et al., 1996). A plausible explanation for this relationship may be that a short birth interval/spacing (which is a constituent of this deprivation index) has a detrimental effect on the health and well-being of both children and their mothers (Goldman and Heuveline, 2000).

Our study reveals that community diversity increase the use of health care services; the more diverse a community, the more the residents of such a community utilize health care services for their sick children. This relationship could be due to many possible reasons for example it may be linked to cultural, educational, and socioeconomic differences between various ethnic groups constituting a given population. In a diverse community, people may be exposed to and adopt new ideas and practices which shape their attitudes to and use of health services.

This present study found that enhanced maternal health seeking behaviour reduced the rates of health services utilization. The likely explanation for this relationship is that it is possible that mothers with high health seeking behaviour in the developing countries receive more preventive interventions, for example they may make sure their children receive immunization (a component of maternal health seeking behaviour) against preventable childhood killer diseases. Their children will thus be less likely to fall ill translating to a reduced usage of health services. Our measure of maternal health seeking behaviour included some basic and important preventive interventions which seem to have a health -promoting or empowering effect thus reducing mothers' need to utilize health service for their children. On the other hand, this statistically significant result might be viewed as counterintuitive and contrary to expectations as use of health services by mothers might imply that greater use of health service might be expected for their children. This finding is however consistent with results of similar studies from developing countries (Uthman, 2009, Goldman and Heuveline, 2000, Abdulraheem and Parakoyi, 2009).

Lastly we found two interaction effects of child's gender, in that the higher the percentage of male children in a community, the greater the effect of the community environmental factor on health service utilization and the more negative the association of maternal health seeking behaviour with health services utilization. In other words, male and female children appear to be dealt with differently in different environmental and behavioural contexts.

## 5 Strengths and limitations

One important limitation of this study is that the available data did not include factors such as ethnocentric practices and sociocultural beliefs, or policies of health authorities and health care delivery systems, all of which may serve as facilitators or barriers to maternal engagement, access to and use of healthcare services. In addition, caution is warranted about drawing conclusions from this cross sectional study because any association at the community level does not or indicate a specific direction of causality and does not guarantee association at the individual level, due to the ecological fallacy (Diez Roux, 2002).

Despite these weaknesses, the study's strengths are significant. The DHS data set has been judged by both international and national health agencies to be the best data available in the developing countries in the absence of viable birth and death registration system. In addition, it is being used extensively for monitoring and evaluation of implemented interventions. To the best of our knowledge, the DHS is the only nationally representative dataset available in most developing countries. Therefore, its use in this study gives credibility to the validity of the study. It is a population based cross sectional study with a large sample size that was randomly selected in order to reflect the true study population. Thus, the findings of this study may be generalizable to other countries with resource-constrained settings in sub-Saharan Africa. In addition, the possibility of selection and sampling bias in this study has been minimized because the whole country's population was considered before implementing multistage random sampling technique for the survey.

### 5.1 Policy implication

This study has provided insights into the factors predicting differences in health services usage for children in Nigerian communities and this will be important information for health policy makers in both governmental and non-governmental organization in understanding the determinants of health service utilization. For example, the findings are relevant in relation to programs encouraging women to use health services (e.g. child spacing services as well as free healthcare for pregnant mothers). Based on our findings, interventions aiming to improve hospital attendance rates for sick children should target communities with an already low rate of health services usage. They should also target communities where there is high multiple childhood deprivation and low community diversity.

## 6 Conclusions

Of the 31,482 children included in this survey; 7577 were sick and 1936 of them were taken to health care facilities for treatment when sick. There are striking variations in health services utilization for sick children across Nigeria communities which appear to be more strongly determined by childhood deprivation factors and maternal health seeking behaviour than by health system functions.

## Authors' contributions

Victor Adekanmbi: Dr. Adekanmbi was involved in the design of the study, data extraction, and statistical analyses, drafting of the article, and interpretation of findings.

Sulaimon Adedokun: Dr. Adedokun was involved in the data extraction, drafting of the article and interpretation of findings.

Sian Taylor-Phillips: Dr. Taylor-Phillips was involved in the drafting of the article and interpretation of findings.

Olalekan Uthman: Dr. Uthman was involved in the design of the study, data extraction, statistical analyses, drafting of the article and interpretation of findings.

Aileen Clarke: Prof. Clarke was involved in the drafting of the article, revising it critically for important intellectual content and interpretation of findings.

All authors approved the final manuscript as submitted and agreed to be accountable for all aspects of the work.

## Funding source

Victor Adekanmbi, Sian Taylor-Phillips, Olalekan Uthman and Aileen Clarke are supported by the NIHR CLAHRC West Midlands initiative. Sulaimon Adedokun was supported by the Consortium for Advanced Research Training in Africa (CARTA) through the fund for his Postdoctoral Fellowship at University of Warwick Medical School. CARTA is jointly led by the African Population and Health Research Center and the University of the Witwatersrand and funded by the Wellcome Trust (UK) (Grant No: 087547/Z/08/Z), the Carnegie Corporation of New York (Grant No: B 8606.R02), Swedish International Development Cooperation Agency (SIDA) (Grant No: 54100029). This paper presents independent research and the views expressed are those of the authors and not necessarily those of the NHS, Wellcome Trust, the NIHR or the Department of Health.

## Conflicts of interest

The authors have no conflicts of interest to disclose.

## Figures and Tables

**Table 1:** Health services utilization for sick children and candidate explanatory variables in Nigeria.

Variable	Mean	SD	Variance
Outcome variable			
Health services utilization per 1000 live births by communities	2.2	2.7	7.1

Community level factors			
Multiple childhood deprivation index^a^	0.0	1.0	
Risky birth interval (%)	17.1	9.2	83.9
High birth order (%)	44.7	16.0	257.2
Low birth weight (%)	13.3	10.3	105.2
Never breastfed (%)	2.6	3.8	14.6
High number of under-fives (%)	9.1	11.5	132.6
Maternal deprivation	0.0	1.0	
Poverty rate (%)	13.0	24.9	621.6
Unemployment rate (%)	26.1	20.0	400.5
Illiteracy rate (%)	33.0	37.1	1376.3
Rural residency (%)	58.6	49.3	2428.9
Community environmental factor index^a^	0.0	1.0	
Safe water source (%)	27.4	32.5	1057.6
Proper sanitation (%)	32.6	32.8	1072.7
Low pollution cooking fuel (%)	2.9	11.5	133.3
Maternal health-seeking behaviour index^a^	0.0	1.0	
Child received at least one vaccination (%)	66.1	19.4	375.6
Ante-natal attendance (%)	16.2	19.4	378.1
Medical assistance at delivery (%)	50.1	35.4	1249.6
Tetanus injection during pregnancy (%)	19.4	19.2	369.7
Ethnicity diversity index^a^	0.0	1.0	
Male gender	17.8	9.4	48.0

a Principal component; SD – standard deviation.

**Table 2:** Predictors of health services utilization by communities in Nigeria – univariable and multivariable models.

Variable	Unadjusted model	Adjusted model
	IRR (95% CI)	IRR (95% CI)
Main effects		
Community factors		
Male gender	1.00 (0.99–1.01)	1.01 (0.99–1.02)
Multiple child deprivation index^a^	1.05 (0.98–1.13)	1.23 (1.12–1.35)
Maternal deprivation index^a^	0.88 (0.82–0.94)	1.06 (0.98–1.33)
Maternal health seeking behaviour index^a^	0.78 (0.73–0.84)	0.64 (0.56–0.73)
Community environmental factor index^a^	1.14 (1.05–1.22)	0.99(0.90–1.08)
Ethnicity diversity index^a^	1.006 (1.003–1.008)	1.005 (1.002–1.007)

Interaction effects		
Multiple child deprivation index^a^ × gender		1.00 (0.99–1.01)
Maternal deprivation index^a^ × gender		1.00 (0.99–1.01)
Maternal health seeking behaviour index^a^ × gender		0.99 (0.98–0.99)
Community environmental factor index^a^ × gender		1.01(1.001–1.02)
Ethnicity diversity index^a^ × gender		1.00 (1.00–1.001)

IRR – incidence rate ratio.

CI – confidence interval.

a Principal component,

## Appendix A Supplementary data

Supplementary tables Image 1
