# Supplementary material for: Predictors of differences in health services utilization for children in Nigerian communities
Source: Prev Med. 2017 Mar;96:67–72. doi: 10.1016/j.ypmed.2016.12.035 (PMC5340469; doi:10.1016/j.ypmed.2016.12.035)
Supplement: Supplementary file 1 — Supplementary tables [file mmc1.doc]

**Supplementary material**

Table 3| Predictors of health services utilisation by communities in Nigeria using backward stepwise model selection – multivariable model

| Variable | IRR (95% CI) | P - value |
| --- | --- | --- |
| **Community factors** |  |  |
| Multiple child deprivation index^a^ | 1.28 (1.18 – 1.39) | <0.001 |
| Maternal health seeking behavior index^a^ | 0.69 (0.64 – 0.75) | <0.001 |
| Ethnicity diversity index^a^ | 1.005 (1.002 – 1.007) | <0.001 |

^a^ Principal component,

IRR – Incidence Rate Ratio,

CI – Confidence Interval

**Table 4**

**Factor analysis/correlation for maternal deprivation index**

| **Factor** | **Eigenvalue** | **Difference** | **Proportion** | **Cumulative** |
| --- | --- | --- | --- | --- |
| Factor 1 | 2.28059 | 1.44667 | 0.5701 | 0.5701 |
| Factor 2 | 0.83392 | 0.21588 | 0.2085 | 0.7786 |
| Factor 3 | 0.61804 | 0.35058 | 0.1545 | 0.9331 |
| Factor 4 | 0.26746 | . | 0.0669 | 1.0000 |

**Table 5**

**Factor analysis/correlation for childhood deprivation index**

| **Factor** | **Eigenvalue** | **Difference** | **Proportion** | **Cumulative** |
| --- | --- | --- | --- | --- |
| Factor 1 | 1.66712 | 0.63538 | 0.3334 | 0.3334 |
| Factor 2 | 1.03174 | 0.09824 | 0.2063 | 0.5398 |
| Factor 3 | 0.93350 | 0.18950 | 0.1867 | 0.7265 |
| Factor 4 | 0.74400 | 0.12037 | 0.1488 | 0.8753 |
| Factor 5 | 0.62363 | . | 0.1247 | 1.0000 |

**Table 6**

**Factor analysis/correlation for maternal health seeking behaviour index**

| **Factor** | **Eigenvalue** | **Difference** | **Proportion** | **Cumulative** |
| --- | --- | --- | --- | --- |
| Factor 1 | 3.18691 | 2.70902 | 0.7967 | 0.7967 |
| Factor 2 | 0.47790 | 0.20588 | 0.1195 | 0.9162 |
| Factor 3 | 0.27201 | 0.20884 | 0.0680 | 0.9842 |
| Factor 4 | 0.06318 | . | 0.0158 | 1.0000 |
